# Terminological Usage Related to Dyspnea by Nursing Staff: A Cross-Sectional Questionnaire Survey

**DOI:** 10.31372/20190404.1065

**Published:** 2020

**Authors:** Yuko Nemoto, Sayuri Suzuki, Shinichiro Okauchi, Katsunori Kagohashi, Hiroaki Satoh

**Affiliations:** a University of Tsukuba-Mito Kyodo General Hospital, Japan; b University of Tsukuba, Mito Medical Center, Japan

**Keywords:** terminological usage, dyspnea, nursing staff, cross-sectional survey, questionnaire

## Abstract

In recent years, additional expressions such as ‘sensation of breathing discomfort’ and ‘discomfort of dyspnea’ are being used in daily nursing care in Japan. To better understand the current status of the use of these terms by nurses, and to ascertain what the term ‘dyspnea’ may not express, we designed an original questionnaire and conducted a study with all nurses at our hospital. The questionnaire included questions to determine if nurses used these terms, and in what context. Of the 279 nurses in our hospital, 225 (80.6%) responded. Three-quarters of nurses indicated that they use these terms in clinical nursing practice. There was no difference in the usage of these terms between nurses who had or had not worked in a respiratory outpatients/ward. However, the percentage of nurses using these terms was higher amongst those with 10 years or less nursing experience compared with those with more than 10 years’ experience. Open-ended questions revealed that these terms were used to communicate information between nurses and between nurses and patients’ families. Our observations need to be verified in large-scale studies to determine if these terms are meaningful for nursing practice in that they describe something not expressed with ‘dyspnea’. There is the possibility of confusion due to the use of inappropriate terms and a lack of education on the subject. Many nurses used these terms, and there may be things that the term ‘dyspnea’ could not express. The results of this study can be used to identify something that is lacking in communication about dyspnea between nurses, nurses and patients, and nurses and patients’ families.

## Introduction

The American Thoracic Society (ATS) defines dyspnea as “a term used to characterize a subjective experience of ‘breathing discomfort’ that is comprised of qualitatively distinct ‘sensations’ that vary in intensity. The experience derives from interactions among multiple physiological, psychological, social, and environmental factors, and may induce secondary physiological and behavioral responses” (ATS, 1999). Dyspnea is the only clinical term that specifically relates to breathing discomfort. However, there are two additional terms commonly used in clinical practice, especially in nursing practice in Japan, that do not currently have a corresponding English expression. One term is ‘kokyu-konnan-kan’ (呼午困難) in Japanese (‘kokyu’ means respiration, ‘konnan’ means difficulty, and ‘kan’ means sensation). This seems like an expression that intentionally adds a “feeling” to the term dyspnea and emphasizes ‘sensation’. It is most closely defined by the ATS expression describing the ‘sensation of breathing difficulty’. The second term is ‘kokyu-ku’ (呼吸苦) in Japanese (‘kokyu’ means respiration, and ‘ku’ means discomfort). This seems to be an expression that intentionally adds “discomfort” to the word dyspnea. The closest ATS expression to this term is ‘discomfort of dyspnea’. These terms are used clinically without a clear definition, and we seem to use these expressions for convenience. Technical terms should be used appropriately. Regarding dyspnea, various terms seem to be used in nursing practice in recent years. This is not limited to our facility, but is the situation broadly in Japan that the Japan Medical Association has identified as a problem (Japan Medical Association, 2015). The background of using other terms instead of the defined term ‘dyspnea’ might be due to the spread of the concept of spiritual pain (Hagmann et al., 2018; Hanson et al., 2017) and the use of expressions of dyspnea with or without objective findings. However, no study has investigated the reason. If it might be a change in expression that takes into account such ideas, this might be a movement that is not limited to Japan. The significance of this study was to help understand the current situation and elucidate the nurses’ own ideas behind the use of these terms. This survey was conducted for two purposes: to ascertain how often these terms are currently being used in practice, and to understand what kinds of meanings were intended by nurses using these terms.

## Methods

### Study Population

A survey of as many nurses as possible in multi-institutional facilities would be ideal. Before conducting multi-institutional research, we conducted a survey at our hospital as a pilot sample study at a tertiary medical institution. This study was approved by the ethical committee of Mito Medical Center, University of Tsukuba-Mito Kyodo General Hospital (Project approval number: NO18-35). A descriptive cross-sectional study using a questionnaire was conducted in March 2019 and included nurses in our hospital. The eligibility criteria were all the nurses working at our hospital, and there were no specific exclusion criteria. All the nurses working at our hospital were invited to participate in the survey.

### Questionnaire

As there was no previous research and no appropriate questionnaire, we developed the original questionnaire described below with some questions requiring a “yes” or “no” response, while other questions allowed an open response (open-ended questions). The questionnaire form contained a section describing the content of the study and an informed consent section. The survey was anonymous and we only included questionnaires for which consent was obtained. The original questionnaire was written in Japanese, but Table 1 shows the English translation of the question and answer section. The main questions were as follows: (1) the nurses’ utilization of these terms, (2) situations in which they use these terms in clinical practice, (3) utilization of these terms in relation to whether the nurse has worked in the respiratory outpatients/ward or not, and (4) utilization of these terms in relation to the extent of their nursing experience. The chief nurse of respiratory outpatients/ward distributed the questionnaires. During the one-week response period, each respondent completed (hand-written) the questionnaire freely at work or at home. Completed questionnaires were collected by the chief nurses and analyzed.

**Table 1:** Questions on ‘sensation of breathing discomfort’ and ‘discomfort of dyspnea’

Questions	
1. Do you use the term ‘sensation of breathing discomfort’?	(Yes, No)
2. Do you use the term ‘discomfort of dyspnea’ when working?	(Yes, No)
3. By whom and where were these terms used? Please describe.	
4. Do ‘dyspnea’, ‘sensation of breathing discomfort’, and ‘discomfort of dyspnea’ mean different things?	(Yes, No)
5. Would ‘sensation of breathing discomfort’ be easier for patients to understand than ‘dyspnea’?	(Yes, No)
6. Is ‘sensation of breathing discomfort’ easier to understand than ‘dyspnea’?	(Yes, No)
7. Can ‘sensation of breathing discomfort’ compensate for something that the term ‘dyspnea’ lacks?	(Yes, No)
8. Can ‘sensation of breathing discomfort’ compensate for something that is not sufficient for ‘dyspnea’?	(Yes, No)
9. Please describe years of nursing experience	_______ (year)
10. Have you worked at respiratory outpatients/ward?	(Yes, No)
11. What do ‘sensation of breathing discomfort’ and ‘discomfort of dyspnea’? Please describe.	

### Statistical Analysis

Data were analyzed with a Chi-squared test with *P* < .05 considered statistically significant.

## Results

### Response to Yes/No Questions

Of the 279 nurses in our hospital, 225 (80.6%) nurses answered the questionnaire. Demographic characteristics of the respondents are shown in Table 2.

**Table 2:** Demographic Characteristics of the Respondents

Total number of nurses in MMC-UT-MKGH	279
Number of respondents	225 (80.6%)
Working experience at the respiratory outpatients/ward	
experienced	120 (53.3%)
not experienced	102 (45.3%)
not answereda	3 (1.3%)
Nursing experiencea	
1–10 years	151 (67.1%)
1–5 years	111 (49.3%)
6–10 years	40 (17.8%)
11 and more years	72 (32.0%)
11–15 years	13 (5.8%)
16–20 years	23 (10.2%)
20 and more years	36 (16.0%)
Not answereda	2 (0.9%)

MMC-UT-MKGH: Mito Medical Center-University of Tsukuba-Mito Kyodo General Hospital.

^a^The answers of nurses who did not respond to years of nursing experience were excluded from this analysis.

In clinical nursing practice, 182 (80.9%) of the 225 nurses answered that they used the term ‘sensation of breathing discomfort’ and 141 (62.7%) used ‘discomfort of dyspnea’. The terms were most often used to express subjective symptoms. The majority of nurses (197; 87.6%) answered that ‘dyspnea’ and ‘sensation of breathing discomfort’ were different, and 129 (57.3%) of nurses felt that ‘sensation of breathing discomfort’ would be easier for patients to understand than ‘dyspnea’. Moreover, while 180 (80.0%) felt that it was better to use both “breathing discomfort” and “breathing difficulty” properly, only 117 (52.0%) of nurses agreed that ‘sensation of breathing discomfort’ is easier to understand than ‘dyspnea’, and only 92 (40.9%) agreed that ‘sensation of breathing discomfort’ compensates for the shortcomings of the term ‘dyspnea’ in communication between nurses.

### Relationship with Years of Nursing Experience

**Figure 1.**
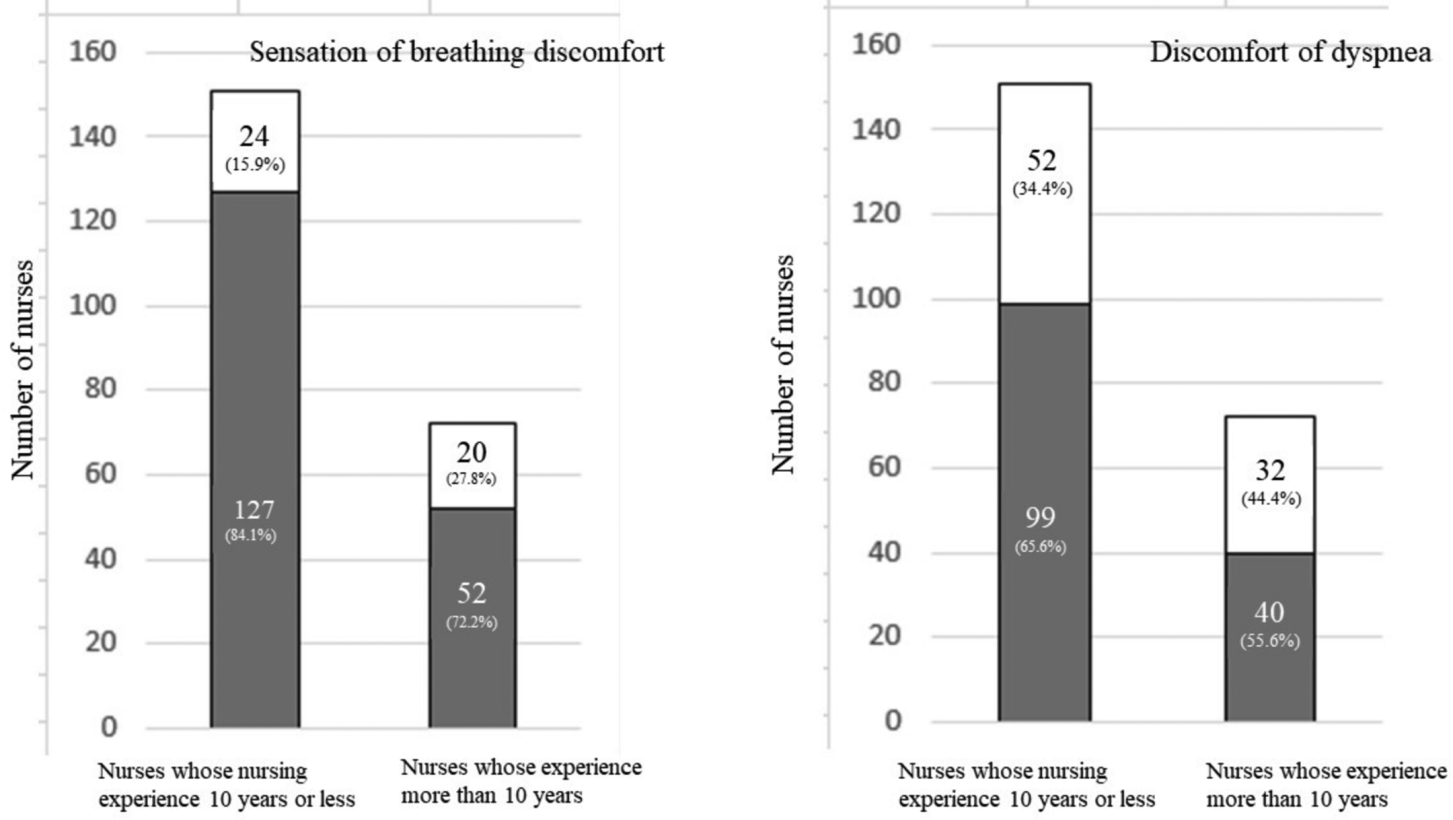
Relationship between years of nursing experience and use of terms related to ‘dyspnea’. Utilization of ‘sensation of breathing discomfort’ and ‘discomfort of dyspnea’ by nurses with 10 years or less of nursing experience compared with those with more than 10 years’ experience. Solid bar: number of nurses who answered ‘Yes, I use the term’; open bar: number of nurses who answered ‘No, I do not use the term’. The percentage of nurses using these terms was higher amongst those with 10 years or less of nursing experience compared with those with more than 10 years’ experience (*P* = .0002 and *P* = .0373, respectively).

Figure 1 shows the utilization of ‘sensation of breathing discomfort’ and ‘discomfort of dyspnea’ by nurses with 10 years or less of nursing experience compared with those with more than 10 years’ experience. Both terms were used significantly more by nurses with 10 years or less of experience (*P* < .0002 and *P* < .0373, respectively).

### Relationship with Respiratory Ward/Outpatient Work

**Figure 2.**
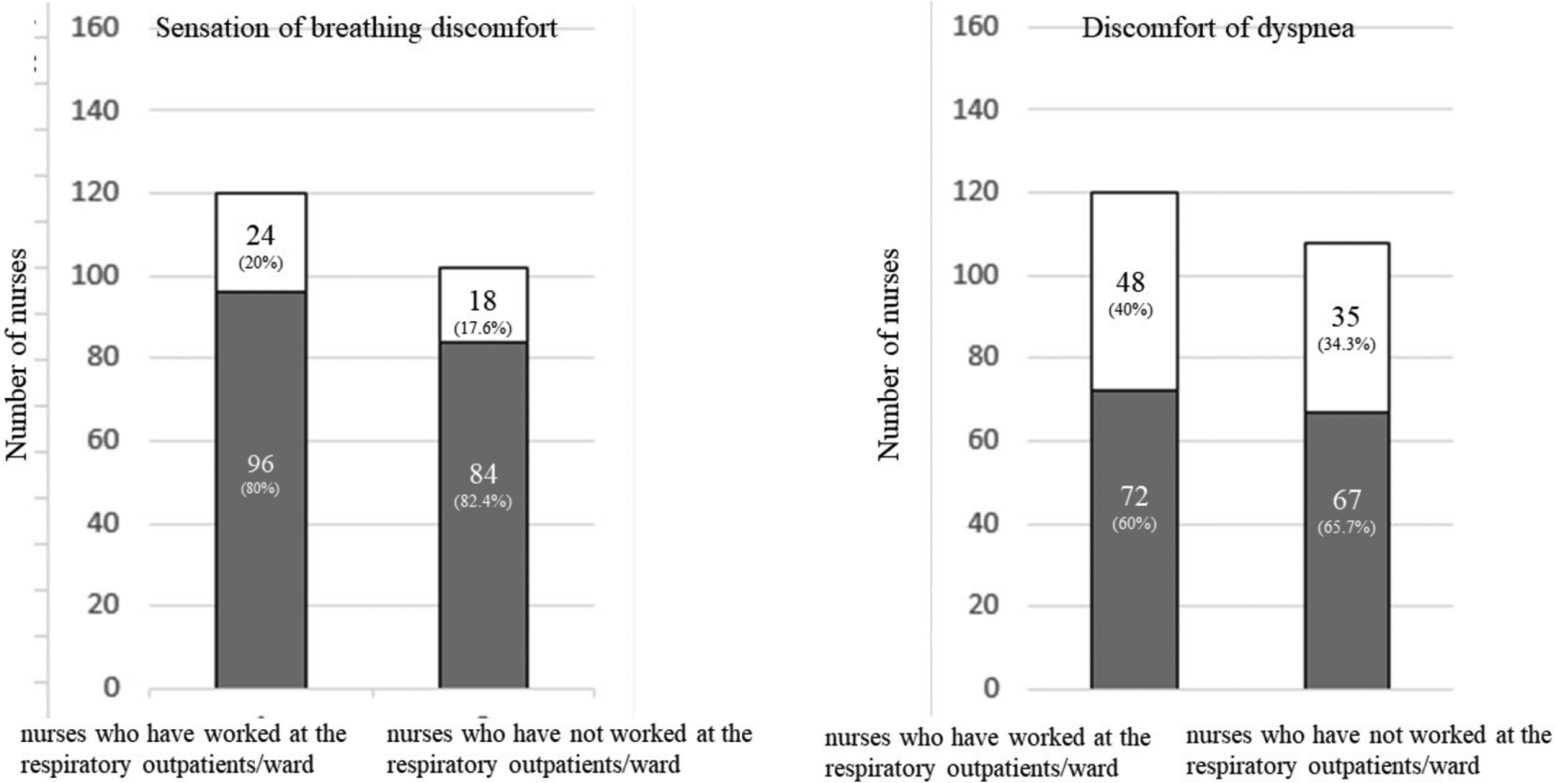
Relationship between respiratory ward/outpatient work experience and use of terms related to dyspnea. Utilization of ‘sensation of breathing discomfort’ and ‘discomfort of dyspnea’ by nurses who have worked in a respiratory outpatients/ward compared with those who have not. Solid bar: number of nurses who answered ‘Yes, I use the term’; open bar: number of nurses who answered ‘No, I do not use the term’. There was no difference in utilization of these terms between the two groups (*P* = .6555 and *P* = .7529, respectively).

Figure 2 shows the utilization of ‘sensation of breathing discomfort’ and ‘discomfort of dyspnea’ by nurses who have worked in the respiratory outpatients/ward compared with those who have not. There was no statistical difference between these two groups (*P* < .6555 and *P* < .7529, respectively).

### Response to Open Questions: By Whom and Where Were These Terms Used?

Nurses mostly used these terms when speaking “nurse to nurse” (172; 76.4%) compared with “nurse to patient family” (13; 5.8%). Regarding the situation these terms were used in, 158 (70.2%) nurses answered “hand over to other nurses” and 29 (12.9%) nurses answered “charting record”. Therefore, these terms are mainly used to communicate information between nurses rather than in explanations to patients’ families.

### Response to Open Questions: What do These Terms Represent?

**Figure 3.**
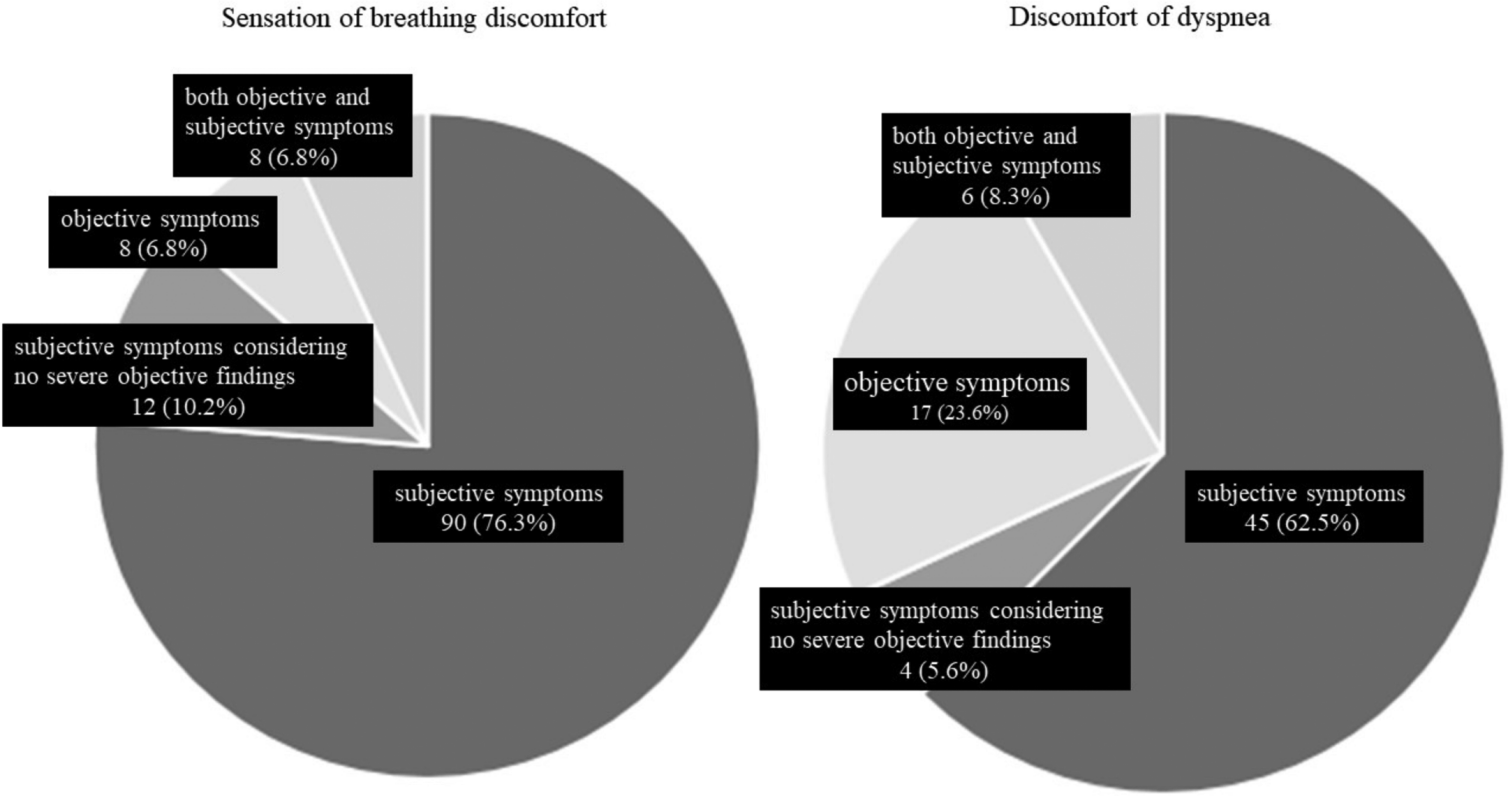
Responses to open questions on ‘sensation of breathing discomfort’ and ‘discomfort of dyspnea’. Ninety (76.3%) of 118 and 45 (62.5%) of 72 nurses, respectively, indicated these terms described ‘subjective symptoms’. Twelve (10.2%) and 4 (5.6%) nurses, respectively, indicated they described ‘subjective symptoms considering no severe objective findings’. Eight (6.8%) and 17 (23.6%), and 8 (6.8%) and 6 (8.3%) nurses indicated the terms described ‘objective symptoms’ or ‘both subjective and objective symptoms’, respectively.

One hundred and eighteen nurses answered what ‘sensation of breathing discomfort’ represented and 72 nurses answered on ‘discomfort of dyspnea’ (Figure 3). Ninety (76.3%) and 45 (62.5%) nurses, respectively, felt these terms describe ‘subjective symptoms’. Interestingly, 12 (10.2%) and 4 (5.6%) nurses, respectively, indicated that these terms describe ‘subjective symptoms considering no severe objective findings’. Eight (6.8%) and 17 (23.6%) nurses, respectively, felt these terms describe ‘objective symptoms’, while 8 (6.8%) and 6 (8.3%) nurses indicated they described ‘both subjective and objective symptoms’.

## Discussion

Three-quarters of nurses in our hospital indicated that they use the terms ‘sensation of breathing discomfort’ and ‘discomfort of dyspnea’ in clinical nursing practice. There was no difference in the usage of these terms between nurses who had or had not worked in a respiratory outpatients/ward. However, the percentage of nurses using these terms was higher amongst those with 10 years or less nursing experience compared with those with more than 10 years’ experience. The two terms were most often used to describe subjective symptoms, but some nurses used them to express ‘objective symptoms’ or ‘both subjective and objective symptoms’. Interestingly, some nurses used these terms to explain subjective symptoms without any accompanying objective findings. Open-ended question revealed that these terms were used to communicate information between nurses and during explanations to patients’ families.

‘Dyspnea’ is a term used to describe subjective symptoms, and the definition by the American Thoracic Society (ATS, 1999) is widely accepted worldwide, including Japan. ‘Dyspnea’ has mainly been studied in patients with cancer (Damani, Ghoshal, Salins, Deodhar, & Muckaden, 2018; Henoch, Bergman, Gustafsson, Gaston-Johansson, & Danielson, 2008; Tanaka, Akechi, Okuyama, Nishiwaki, & Uchitomi, 2000) and COPD (Anzueto & Miravitlles, 2017; Hanania & O’Donnell, 2019; Soffler, Hayes, & Schwartzstein, 2017), especially those with terminal stage respiratory failure (Hashimoto, Yoshida, & Kanda, 2017; Pisani, Hill, Pacilli, Polastri, & Nava, 2018). In physiology, some researchers refer to dyspnea as ‘sensation of breathing discomfort’ to emphasize not only the clinical symptoms, but also sensory perception (Anzueto & Miravitlles, 2017; Dangers et al., 2017; O’Donnell, Milne, Vincent, & Neder, 2019). These terms seem to be used in routine clinical practice and have appeared in several publications in our country (Hashimoto et al., 2017; Ito, Imura, & Takaku, 2009; Wada, Minami, & Komine, 2010). However, there is no research on the present conditions of their usage. In addition, there is no formal definition of the meaning or usage of these terms. In 2015, the term committee of the Japan Medical Association subcommittee discussed the use of the terms ‘sensation of breathing discomfort’ and ‘discomfort of dyspnea’, and concluded they were not proper medical terms (Japan Medical Association, 2015). However, these terms are frequently used despite being regarded as inappropriate medical terms. In the field of nursing studies in recent years, there has been vast progress in research on ‘pain’ such as ‘total pain’ (Abernethy & Wheeler, 2008; Saunders & Baines, 1983; Tan et al., 2017), ‘spiritual pain’ (Hagmann et al., 2018; Hanson et al., 2017), and ‘total dyspnea’ (Abernethy & Wheeler, 2008; Kamal, Maguire, Wheeler, Currow, & Abernethy, 2011; Lovell, Etkind, Bajwah, Maddocks, & Higginson, 2019). In such situations, if medical staff feel ‘something’ which cannot be expressed by the term ‘dyspnea’ in many aspects of medical practice and seek alternative expressions, this may not be a situation unique to Japan. Given these circumstances, we considered it worthwhile to understand the current state of the use of these terms. Our study clarified some aspects.

The only defined term to indicate the subject’s symptoms is ‘dyspnea’. The results of this study indicate there may be a lack of nursing education regarding the proper use of this clinical term. Furthermore, in the medical and nursing fields, the term ‘dyspnea’ is often (carelessly or intentionally) translated into Japanese as ‘sensation of breathing discomfort’ and ‘discomfort of dyspnea’ (Hashimoto et al., 2017; Ito et al., 2009; Wada et al., 2010). Thus, in addition to correct use of the terms, there are likely to be variations in their use in different languages. However, the reason may not only be a difference in language. If there is a need in clinical nursing practice where ‘sensation of breathing discomfort’ and ‘discomfort of dyspnea’ can describe ‘something’ not conveyed by ‘dyspnea’, they may need to be clarified and defined as clinical terms.

This study has some limitations. First, we composed the questionnaire ourselves due to a lack of appropriate published surveys, and it is possible it had design flaws. Second, since this was a study of a small number of nurses at a single medical institution, we cannot make any general conclusions. Third, as neither ‘sensation of breathing discomfort’ nor ‘discomfort of dyspnea’ have appropriate English expressions, they may not be able to be translated adequately into English. Despite these potential or real caveats, our results help to understand the current situation and highlight associated issues.

It is important for every nurse to support patients with appropriate communication. We have reported the present study to raise awareness of the current usage of terms related to dyspnea and the need for expressions other than ‘dyspnea’. Language and clinical practice are different among Asian/Pacific Islanders, and it is important to understand them. The role of nurses to understand and support the patient’s suffering is important, even if they have different backgrounds. It is desirable to accurately understand subjective symptoms with universal expressions that transcend these differences. Without curtailing use as inappropriate due to undefined terms, it might be meaningful to examine the background of these terms that have begun to be used voluntarily among nurses.

## Conclusions

Our results showed that the majority of nurses used the terms ‘sensation of breathing discomfort’ and ‘discomfort of dyspnea’. A discussion of the adequacy of term use was not performed, but we do know each nurse used these terms with intention. It will be necessary to verify these results in large-scale studies to determine if there is ‘something’ that cannot be expressed with ‘dyspnea’ and if these terms have meaning in nursing practice.

## Author Contributions

Y.N., S.S., and H.S. designed the study. Y.N., S.S., and H.S. created the questionnaire. Y.N. and S.S. collected the data. Y.N., S.O., K.K., and H.S. analyzed the data. Y.N., S.O., K.K., and H.S. prepared the manuscript. All authors approved the final version for submission.

## Declaration of Conflicting Interests

The authors declared no potential conflicts of interest with respect to the research, authorship, and/or publication of this article.

## Funding

None declared.

## Authorship Statement

All authors met the authorship criteria and were in agreement with the content of the manuscript.

## References

[R1] Abernethy A. P., & Wheeler J. L. (2008). Total dyspnoea. *Current Opinion in Supportive and Palliative Care*, 2 (2), 110–113. 10.1097/SPC.0b013e328300cad0 18685406

[R2] American Thoracic Society. (1999). Dyspnea mechanisms, assessment, and management: A consensus statement. *American Journal of Respiratory and Critical Care Medicine*, 159 (1), 321–340. 10.1164/ajrccm.159.1.ats898 9872857

[R3] Anzueto A., & Miravitlles M. (2017). Pathophysiology of dyspnea in COPD. *Postgraduate Medicine*, 129 (3), 366–374. 10.1080/00325481.2017.1301190 28277858

[R4] Damani A., Ghoshal A., Salins N., Deodhar J., & Muckaden M. (2018). Prevalence and intensity of dyspnea in advanced cancer and its impact on quality of life. *Indian Journal of Palliative Care*, 24 (1), 44–50. 10.4103/IJPC.IJPC_114_17 29440806 PMC5801629

[R5] Dangers L., Laviolette L., Georges M., Gonzalez-Bermejo J., Rivals I., Similowski T., & Morelot-Panzini C. (2017). Relieving dyspnoea by non-invasive ventilation decreases pain thresholds in amyotrophic lateral sclerosis. *Thorax*, 72 (3), 230–235. 10.1136/thoraxjnl-2016-208544 27507899

[R6] Hagmann C., Cramer A., Kestenbaum A., Durazo C., Downey A., Russell M., … Roeland E. J. (2018). Evidence-based palliative care approaches to non-pain physical symptom management in cancer patients. *Seminars in Oncology Nursing*, 34 (3), 227–240. 10.1016/j.soncn.2018.06.004 30120000

[R7] Hanania N. A., & O’Donnell D. E. (2019). Activity-related dyspnea in chronic obstructive pulmonary disease: Physical and psychological consequences, unmet needs, and future directions. *International Journal of Chronic Obstructive Pulmonary Disease*, 14, 1127–1138. 10.2147/COPD.S188141 31213793 PMC6538882

[R8] Hanson L. C., Collichio F., Bernard S. A., Wood W. A., Milowsky M., Burgess E., … Lin F. C. (2017). Integrating palliative and oncology care for patients with advanced cancer: A quality improvement intervention. *Journal of Palliative Medicine*, 20 (12), 1366–1371. 10.1089/jpm.2017.0100 28737996 PMC5749575

[R9] Hashimoto H., Yoshida K., & Kanda K. (2017). Concept analysis of a feeling of dyspnea among cancer patients. *Journal of Japanese Society of Nursing Research*, 40, 45–56 10.15065/jjsnr.20161107005

[R10] Henoch I., Bergman B., Gustafsson M., Gaston-Johansson F., & Danielson E. (2008). Dyspnea experience in patients with lung cancer in palliative care. *European Journal of Oncology Nursing*, 12, 86–96. https://www.ncbi.nlm.nih.gov/pubmed/18023256 18023256 10.1016/j.ejon.2007.09.006

[R11] Ito M., Imura H., & Takaku F. (ed.) (2009). *The Encyclopedia of medicine* (2nd ed.). 959, Tokyo: Igakushoin (Original work published in Japanese).

[R12] Japan Medical Association. (2015). *Proceedings of the Term Committee of the Japan Medical Association Subcommittee*, 9–10 (Original work published in Japanese).

[R13] Kamal A. H., Maguire J. M., Wheeler J. L., Currow D. C., & Abernethy A. P. (2011). Dyspnea review for the palliative care professional: Assessment, burdens, and etiologies. *Journal of Palliative Medicine*, 14 (10), 1167–1172. 10.1089/jpm.2011.0109 21895451 PMC3189385

[R14] Lovell N., Etkind S. N., Bajwah S., Maddocks M., & Higginson I. J. (2019). Control and context are central for people with advanced illness experiencing breathlessness: A systematic review and thematic synthesis. *Journal of Pain and Symptom Management*, 57 (1), 140–155.e2. 10.1016/j.jpainsymman.2018.09.021 30291949

[R15] O’Donnell D. E., Milne K. M., Vincent S. G., & Neder J. A. (2019). Unraveling the causes of unexplained dyspnea: The value of exercise testing. *Clinics in Chest Medicine*, 40 (2), 471–499. 10.1016/j.ccm.2019.02.014 31078223

[R16] Pisani L., Hill N. S., Pacilli A. M. G., Polastri M., & Nava S. (2018). Management of dyspnea in the terminally ill. *Chest*, 154 (4), 925–934. 10.1016/j.chest.2018.04.003 29679597

[R17] Saunders C., & Baines M. (1983). *Living and dying the management of terminal disease*. Oxford Medicine Publications.

[R18] Soffler M. I., Hayes M. M., & Schwartzstein R. M. (2017). Respiratory sensations in dynamic hyperinflation: Physiological and clinical applications. *Respiratory Care*, 62 (9), 1212–1223. 10.4187/respcare.05198 28655742

[R19] Tan J. Y., Yorke J., Harle A., Smith J., Blackhall F., Pilling M., & Molassiotis A. (2017). Assessment of breathlessness in lung cancer: Psychometric properties of the Dyspnea-12 Questionnaire. *Journal of Pain and Symptom Management*, 53 (2), 208–215. 10.1016/j.jpainsymman.2016.08.009 27720789

[R20] Tanaka K., Akechi T., Okuyama T., Nishiwaki Y., & Uchitomi Y. (2000). Development and validation of the Cancer Dyspnoea Scale: A multidimensional, brief, self-rating scale. *British Journal of Cancer*, 82 (4), 800–805. https://www.ncbi.nlm.nih.gov/pmc/articles/PMC2374383/pdf/82-6691002a.pdf 10732749 10.1054/bjoc.1999.1002 PMC2374383

[R21] Wada O., Minami Y., & Komine M. (ed.) (2010). *Nursing dictionary* (2nd ed.). 1081, Tokyo: Igakushoin (Original work published in Japanese).

